# Evaluation of the CompreHensive geriAtRician-led MEdication Review (CHARMER) deprescribing intervention in hospital: protocol for a cluster randomised stepped-wedge trial

**DOI:** 10.1136/bmjopen-2025-107396

**Published:** 2026-03-18

**Authors:** David John Wright, David Phillip Alldred, Sion Scott, Bethany Atkins, Allan B Clark, Antony Colles, Amber Hammond, Charlotte E L Jones, Jacqueline M Martin-Kerry, Martyn Patel, Erika Sims, David Turner, Miles Witham, Debi Bhattacharya

**Affiliations:** 1 School of Healthcare, College of Life Sciences, University of Leicester, Leicester, UK; 2 School of Healthcare, University of Leeds, Leeds, UK; 3 Bradford Institute for Health Research, NIHR Yorkshire and Humber Patient Safety Research Collaboration, Leeds, UK; 4 School of Health Sciences, Faculty of Medical and Health Sciences, University of East Anglia, Norwich, Norfolk, UK; 5 Norwich Clinical Trials Unit, Norwich Medical School, University of East Anglia, Norwich, UK; 6 Norfolk and Norwich University Hospital, Norwich, UK; 7 NIHR Newcastle Biomedical Research Centre, Newcastle University, Newcastle upon Tyne, UK

**Keywords:** Hospitals, Pharmacists, GERIATRIC MEDICINE, Medication Review, Behavior

## Abstract

**Background:**

While almost half of older adults admitted to hospital are prescribed potentially inappropriate medicines, less than 1% have a medicine proactively deprescribed during admission in the UK. The CompreHensive geriAtRician-led MEdication Review (CHARMER) intervention is designed to address geriatricians’ and pharmacists’ barriers and enablers to deprescribing. The CHARMER definitive trial will evaluate effectiveness, cost-effectiveness and safety.

**Methods:**

A stepped-wedge cluster randomised controlled trial will be conducted in 20 hospitals in England, with four hospitals in reserve. All hospitals will collect baseline data. Every 3 months, five hospitals will be randomised to receive the intervention. The intervention, implemented by a local project manager, comprises a hospital action plan to set deprescribing as an organisational goal; workshops for pharmacists and geriatricians to change beliefs about deprescribing; weekly briefings between geriatricians and pharmacists to discuss opportunities for deprescribing; benchmarking reports to compare deprescribing performance across participating hospitals. With an average of 200 patients admitted and discharged during each step, the study will have 89.5% power at 5% significance level and intra-class correlation coefficient of 0.05 to detect a 3% difference in 90-day re-admission rate from 16.7% versus 13.7%. Anonymised routinely collected data, including readmissions, will be obtained for all patients admitted during the study period. Enhanced data collection periods of 1 month during control and intervention periods will be used to recruit patients and data for secondary outcomes and process evaluation.

**Discussion:**

A stepped-wedge design enabled a smaller number of hospitals and patients to be included than a traditional cluster-randomised design. The complexity of intervention implementation necessitated a project manager in addition to the principal investigator responsible for trial conduct. Using routinely collected data for the primary outcome measure should ensure that the trial has sufficient power on completion. Planned enhanced data collection for short periods of time improves trial efficiency.

**Trial registration number:**

ISRCTN13248281.

**STRENGTHS AND LIMITATIONS OF THIS STUDY**

- Cluster-randomised stepped-wedge design trial.
- Large multi-site hospital-based deprescribing trial in older person’s setting.
- Feasibility-tested trial and theory-informed intervention.
- Collation of primary outcome measure for all patients recruited onto the ward to optimise likelihood of achieving target sample size.
- Enhanced data collection period to obtain secondary outcome data and inform process evaluation.

## Introduction

### Background and rationale

 The WHO Global Patient Safety Challenge: Medication Without Harm aims to reduce severe avoidable medication-related harm by 50%.^1^ Polypharmacy, as a risk factor for both patient harm^2^ and death,^3^ is recognised as a ‘key action area’ by the WHO.^1^ A UK national review of prescribing in 2021 recognised the significant extent of overprescribing and recommended several primary care-based interventions to address this.^4^ A meta-analysis of deprescribing interventions in primary care identified a small reduction in all-cause mortality, thereby justifying the recommendation.^5^

While it is estimated that half of older people at the point of entering hospital are already prescribed at least one medicine which may be potentially inappropriate,^6^ very few are stopped during admission in the UK.^7^ When deprescribing does occur, it is usually in response to presenting harm, that is, it is reactive rather than proactive.^7^ An opportunity to reduce overprescribing in hospital through proactive deprescribing is therefore being missed and may supplement similar activities in primary care. Hospital deprescribing trials have historically focused on process measures such as reduction in the proportion of potentially inappropriate medicines, rather than meaningful clinical outcomes.^8^

A lack of focus on addressing the barriers and enablers to deprescribing in hospitals when developing interventions may explain the lack of efficacy reported in existing deprescribing trials.^8 9^ We have used behaviour change theory to underpin the development of an intervention that is designed to address geriatricians’ and pharmacists’ capability, opportunity and motivation to identify opportunities to deprescribe and initiate deprescribing discussions with older people. Combined with extensive co-design work with the target audiences,^10^ our approach may enhance the likelihood of good implementation fidelity and resulting effectiveness.^11^

We developed the CompreHensive geriAtRician-led MEdication Review (CHARMER) deprescribing intervention using evidence regarding the barriers and enablers to deprescribing and behaviour change theory.^10 12 13^ CHARMER aims to facilitate geriatricians and pharmacists to identify opportunities for proactive deprescribing and initiate deprescribing conversations with older adults in hospital. The intervention comprises five components:

- A hospital action plan to set deprescribing as an organisational goal to be achieved by implementing the remainder of the CHARMER intervention components.
- A geriatrician workshop with pre-prepared videos of peers navigating challenging deprescribing discussions with patients and relatives.
- A pharmacist workshop with case studies to address negative beliefs about the consequences of deprescribing.
- Weekly pharmacist and geriatrician briefings to identify opportunities for proactive deprescribing.
- Weekly benchmarking reports on deprescribing activities to allow sites to compare their performance against other sites.

In line with the UK Medical Research Council guidance for developing and evaluating complex interventions,^14^ the CHARMER intervention and trial design were feasibility tested.^15^ This led to numerous changes to both intervention implementation support and trial design, with the most significant being the decision to move from a cluster RCT to a cluster randomised stepped-wedge design. Due to the increase in power associated with a stepped-wedge design, this change requires half the number of hospitals for the trial representing more efficient trial design and thus better value for money. Furthermore, this design provides longer term follow-up data in a proportion of intervention hospitals and will thereby allow us to assess whether the intervention is normalised.

### Objectives

To describe the design of the definitive trial of the CHARMER hospital deprescribing intervention to evaluate its effectiveness, cost-effectiveness and safety.

### Trial design

Cluster randomised stepped-wedge design trial with internal pilot. A maximum of 24 hospitals (20 active, four reserve) across England will be recruited to take part. All participating hospitals will begin the trial at the same time in a control phase.

**Figure 1:**
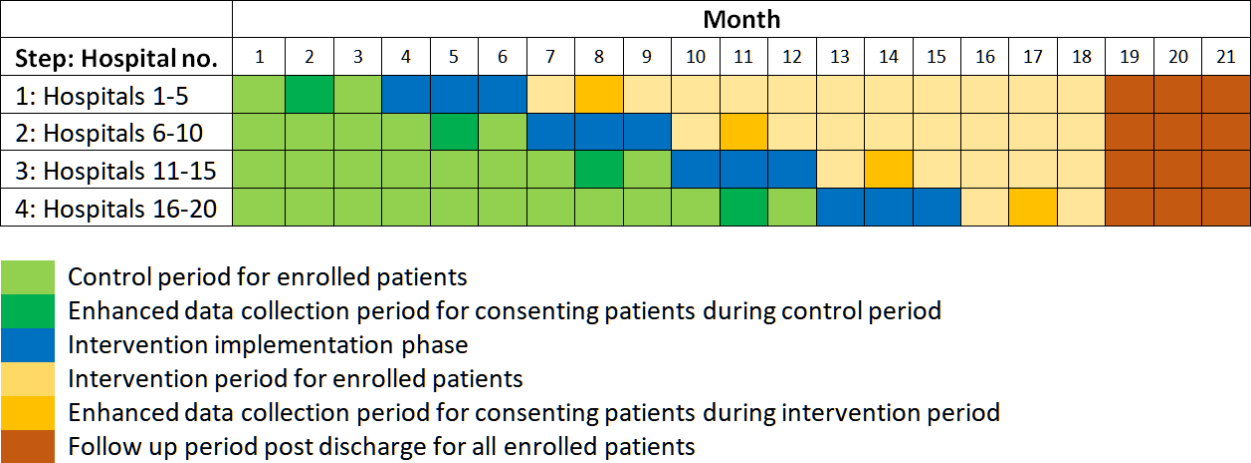
Schedule of enrolment, consent, intervention and data collection.

We will step hospital sites into the intervention ‘implementation phase’ at 3 month intervals (five hospitals every 3 months in four steps). The intervention ‘implementation phase’ will last for 3 months for all hospitals, prior to transferring into the active intervention phase. Each site will therefore take part in the trial for a total of 18 months (with an additional 3-month follow-up period for data collection). This is summarised in figure 1.

To ensure effective implementation of the intervention, a Project Manager is required at each site. This will usually be in addition to the Principal Investigator (PI), who is responsible for research delivery. Where capacity allows, the PI will be allowed to additionally assume the role of Project Manager.

Outlined here is the protocol for the main trial. Process evaluation protocol and results papers will be published separately.

## Methods: participants, interventions and outcomes

Methods outlined below based on Protocol V.1.1 2023. The trial is registered with ISRCTN Registry 13 248 281 University of Leicester (rgosponsor@le.ac.uk) acted as sponsor for the trial. They took no active involvement in either the management or delivery of the trial.

### Patient and public involvement

A patient and public involvement (PPI) group consisting of older adults experiencing polypharmacy (n=3) and family members/carers (n=2) are core members of the CHARMER research team. Our PPI group has contributed to the development and design of this trial, including developing the protocol, reviewing and editing participant information sheets and consent forms to ensure readability, and commenting on qualitative topic guide content. PPI group members attend weekly trial management meetings and will support the research team in the analysis, write-up and dissemination of the study findings.

### Trial setting

Acute hospitals in England with care of the older wards.

### Eligibility criteria

Proposed inclusion and exclusion criteria for hospitals, practitioners and patients are:

### Hospitals

Inclusion

- Acute hospital.
- Provide an older people’s medicine service with at least one inpatient ward on which to conduct the CHARMER trial (the study ward).
- The majority of patients on the study ward(s) have a minimum length of stay of 3 days.
- The average total monthly throughput across study ward(s) of approximately 100 patients has at least one geriatrician (consultant or specialist registrar) and at least one ward-based pharmacist working on them.
- There is an electronic prescribing system on the study ward(s).
- The hospital is willing and has the capability and capacity to implement the five components of the CHARMER intervention.
- Able to provide a 0.2 whole time equivalent Project Manager to oversee implementation of the CHARMER intervention over a 14-week period (3 month implementation phase and 2 weeks within the active intervention phase).
- Named clinician willing and appropriate to take PI responsibility.
- Suitably trained staff, for example, sufficient research nurses, to recruit patient and personal consultee participants and enter required data.

Exclusion

- Wards that primarily host patients who are medically fit for discharge and awaiting discharge care packages.
- Hospitals without a ward-based pharmacy service.
- Mental health hospitals.
- Hospitals that implemented the intervention in the feasibility study.

### Practitioners

Inclusion criteria:

- Consultant geriatrician or specialist registrar and appropriately qualified pharmacists working across Older People’s Medicine wards.

Exclusion criteria:

- Less than 0.3 whole time equivalent of ward-based time for pharmacists.

### Who will take informed consent?

Hospitals will be recruited to participate through the National Institute for Health and Care Research (NIHR) Research Delivery Network (RDN) in England.^16^ Funding to incentivise participation within hospitals is provided nationally and based on the number of enrolled patients. Consent for geriatricians and pharmacists to participate will be taken by the central research team within the Norwich Clinical Trials Unit (NCTU).

Consent for patients to provide non-routinely collected data for the enhanced data collection periods will be undertaken by local research nurses or members of the healthcare team with the relevant training in obtaining informed consent for research purposes. These individuals will usually be employed by the RDN. Any patients deemed inappropriate by the research nurses or members of the healthcare team, such as those lacking capacity or near end of life, will not be consented.

### Interventions

#### Explanation for the choice of comparators

The control steps will consist of the provision of usual care for older people admitted to participating older people’s medicine wards.

#### Intervention description

The intervention is summarised in the background and described in detail in a separate publication.^10^ The action plan and geriatrician and pharmacist workshops will be delivered during the 3-month implementation phase. The weekly briefings and benchmarking reports will occur throughout the active intervention phase.

#### Criteria for discontinuing or modifying allocated interventions

There are no plans to discontinue or modify any elements of the intervention as the trial progresses.

#### Strategies to improve adherence to the intervention

The intervention will be delivered to geriatricians and pharmacists (the target audience) and not patients. The Project Managers have been employed to ensure that the intervention is implemented as intended and in alignment with the Trust’s standard procedures. Once the trial is complete, hospitals and participating practitioners can decide whether they wish to continue with the intervention.

#### Relevant concomitant care permitted or prohibited during the trial

The intervention is focused on healthcare professionals with an increase in deprescribing discussions and activity an anticipated outcome. Patients will continue to receive all types of concomitant care during the trial with nothing prohibited.

#### Provisions for post-trial care

There are no provisions for post-trial care. Communication of any medicines deprescribed to primary care physicians will be undertaken as part of standard care at time of hospital discharge.

### Outcomes

Selection of outcome measures was based on a previously developed core outcome set designed for hospital-based deprescribing interventions.^17^ The primary outcome measure was selected as hospital readmission within 90 days as this was considered to be sufficiently sensitive and proximal to the intervention.

Outcome data are summarised in table 1. Costs and resource use, as well as economic outcomes, will be captured via expert opinion and review of study records where relevant.

**Table 1:** Outcome data

Routinely collected data (without consent)	When	How
Mortality for any reason	90 data post-discharge	Hospital records and NHS England* Office of National Statistics Mortality records
Number of hospital stays†	90 data post-discharge	Hospital records and NHS England* Hospital Episode Statistics Admitted patient care
Number of planned and unplanned admissions and readmissions for treatment or monitoring of health†		

Intensive data collection (with consent)¶	When	How
Satisfaction with deprescribing process‡	2 weeks post-discharge	Postal questionnaire
Adverse drug events§	90 days post-discharge	Telephone questionnaire
Health-related quality of life (EQ-5D-5L††)^23^	Pre-discharge 90 days post-discharge	Face to face on ward Telephone questionnaire
Primary care consultations†	90 days post-discharge	Telephone questionnaire
Community pharmacy dispensed medicines†	90 days post-discharge	NHS England*
Number of ‘Regularly’ prescribed medicines	On admission and discharge	Routine hospital records^**^
Number of ‘As required’ prescribed medicines		
Number of medicines prescribed on admission which are stopped during admission while on study ward		
Number of medicines prescribed on admission with dosage reduced during admission while on study ward		
Number of stopped medicines prescribed on admission which are stopped during admission while on study ward and then restarted		

* National agency responsible for collation and provision of healthcare data.

† Captured for additional health economic analysis purposes.

‡ Questionnaire with 12 items based on previously published survey tool.^25^

§ Questionnaire with 17 items inviting respondents to indicate whether or not they have experienced any of the medication-related adverse events listed. The list is derived from an observational study of medication-related adverse events.^26^

¶ During enhanced data collection periods, consent/assent for routine data collection was sought wherever possible, but where not possible, Section 251 approval was applied. Where participants declined to consent/assent, routine data was not collected.

** To enable the outcomes regarding medication to be collated the following data will be collected: Name, dose and frequency of all medicines prescribed to the patient before any changes were made during the hospital admission. Any changes made to the above medicines (stopped, dose reduction, dose increase or change in frequency). Name, dose and frequency of any new medicines started during the admission that are requested to be continued post discharge.

†† EQ-5D-5L, EuroQol 5-Dimension 5-Level.

NHS, National Health Service.

Geriatrician and pharmacist participants will complete the following at the point of site set up:

- Short demographics questionnaire (age, gender, ethnicity, job role, grade, training).
- Mechanism of action questionnaire—an 11-item questionnaire measuring the extent to which the CHARMER intervention has addressed the intended four barriers and one enabler to proactive deprescribing.^15^
- Process of deprescribing questionnaire—a 19-item questionnaire capturing the extent to which practitioners undertake the steps within the process of proactively deprescribing in hospital.^18^

### Withdrawal

Hospitals, geriatricians and pharmacists and patient participants will be able to withdraw from the study at any time, without providing a reason, by informing a member of the research team. If a hospital chooses to withdraw from the trial, all data collected up until the point of withdrawal will be retained and a replacement site may be recruited.

If geriatrician and pharmacist participants choose to withdraw during the trial, any data they have already contributed as participants will continue to be used. If it is feasible and the trial can progress as planned, we will work with the relevant PI to recruit a replacement geriatrician or pharmacist if one withdraws. If this is not possible, for example, study is nearing completion, we will continue the trial with the remaining geriatricians and pharmacists.

Where patients choose to withdraw, although not obliged to give a reason, a reasonable effort will be made to establish this reason, while remaining fully respectful of the participant’s rights. All study data collected up to the point of withdrawal will be retained.

### Internal pilot

An internal pilot will be conducted with Step 1 hospitals with the following stop/go criteria:

- An average of 200 patients per hospital enrolled over 3 months (Green).
- An average of 150–199 patients per hospital enrolled over 3 months (Amber).
- Below an average of 150 patients per hospital enrolled over 3 months (Red).

If ‘Green’, the trial will proceed. ‘Amber’ it will proceed if appropriate solutions to improve recruitment are identified. ‘Red’ will require the Trial Steering Committee to make a decision regarding whether to proceed.

### Participant timeline

Hospitals will participate in the trial from month 0 to month 21. Hospitals will be required to identify a PI responsible for local trial delivery and project manager for intervention implementation. Geriatricians and pharmacists will participate during the intervention implementation period until trial completion.

All patients admitted to the study wards under the care of a participating geriatrician will be enrolled, with permission from the national Confidentiality Advisory Group (CAG), for inclusion of their routinely collected data. This is unless they have notified the NHS in advance of a desire to be excluded from all research activity.

Figure 1 shows how patients will be enrolled into the trial, separating out control, implementation and intervention periods. The implementation phase is the point at which the intervention is delivered to the participating doctors and pharmacists, such that post-implementation, the intervention can be delivered.

During ‘enhanced data collection periods’, patients consenting to participation on a study ward under the care of participating geriatricians will be participants from the time of admission until 3 months post-discharge. We will notify the consented patient’s primary care medical practice by letter of their participation.

### Site set up

We will provide the Project Manager and PI with a CHARMER intervention implementation handbook, developed for the feasibility study and subsequently refined.^15^ A CHARMER trial handbook, comprising instructions regarding delivering the trial and CHARMER intervention in accordance with the approved research ethics and governance processes, will be provided to the PI.

The site will be expected to conduct the trial in compliance with the approved research protocol. The PI or their delegate will be required to document and explain any deviation from the approved protocol and communicate this to the study team at NCTU.

### Sample size

The underpinning feasibility study showed that recruitment to an original planned cluster randomised trial would require recruitment of over 40 hospitals and was unlikely to be achieved. Consequently, the design was changed to a stepped wedge due to the increased power provided by this design.

With a baseline step with all hospitals receiving the control followed by four separate steps, each randomising five hospitals to switch from control to intervention (20 hospitals in total) will have 89.5% power to detect a difference of 16.7% to 13.7% of 90-day re-admission rates assuming a 5% level of significance and an intra-class correlation coefficient (ICC) of 0.05 and 200 participants per hospital per step. The total sample size will be 20 000 participants. The anticipated 3% difference is similar, but slightly smaller, to the difference reported in the MedSafer study.^19^

From our feasibility study findings, we estimate that 10% of patients on study wards or consultees will provide written (or, if required, virtual) informed consent for additional patient-reported outcome data. For the enhanced data collection period, where medication changes are recorded in hospital, then if the sites collect data on 60 participants in each of the enhanced data collection periods, the study will have 99.99% power to detect an average change of 0.5 medications between the control and intervention phase, assuming a SD of 1.5 medications, a 5% level of significance and an ICC of 0.05.

### Recruitment

All patients receiving treatment under the care of participating geriatricians on the study wards within the study window (1 February 2024 to 31 July 2025) will be eligible for inclusion.

With national CAG^20^ permission, all patients admitted to wards under the care of participating consultant geriatricians will be enrolled for routine data collection purposes, enabling collation of the primary outcome measure for all patients. Consequently, we will not be required to enact any strategies for achieving adequate participant enrolment.

During enhanced data collection periods, 1 month during the control period and 1 month during the intervention period, patients admitted to wards under the care of participating consultant geriatricians will be approached to be consented for enhanced data collection, that is, non-routinely collected data needed for secondary outcome measures and process evaluation purposes.

### Assignment of interventions: allocation

#### Sequence generation

Sites will be randomised to the four ‘groups’, the ‘groups’ being the step at which they started the intervention. Group 1 will start the intervention on the first step, etc. A fifth group, Group 5, is the reserve list. To ensure no two sites from the same region are randomised to the same group and thereby overwhelm the local RDN team, randomisation will be stratified by region. A block size of 4 will be used so that randomisation could occur once four sites are recruited and concealment maintained.

#### Concealment mechanism

Randomisation will occur after every fourth site is recruited. If sites from the same region are participating, randomisation will be delayed until they can all be randomised at the same time. This process ensures concealment as the recruitment team will be unable to predict treatment allocation.

#### Implementation

The list will be generated using the Sealed Envelope online randomisation list generator. Sites will be recruited and once four sites confirm capability and capacity to deliver the research, they will be allocated to an intervention step by the trial manager using the REDCap^21^ randomisation system.

#### Blinding

Due to the nature of the intervention and trial design, blinding to allocation is not possible.

### Data collection and management

#### Plan for assessment and collection of outcomes

We will collect relevant outcome data at two levels for patients admitted to study wards and under the care of participating consultant geriatricians:

1. Routinely collected data for all patients admitted for the duration of control and intervention steps, that is, 18 months.
2. Non-routinely collected data during ‘enhanced data collection periods’ which take place for 4 weeks during both the control and intervention period.

Routine data will be collected by nurses employed within the national RDN^16^ for all patients under the care of a participating geriatrician admitted to the study wards during the trial period and provided anonymously to the research team following national CAG^20^ permission to collect these data without the requirement for patient consent.

#### Plans to promote participation retention and complete follow-up

With enrolment of all patients and collation of routinely collected data, there are no plans to promote participation retention for the purposes of the primary outcome measure.

Due to the short duration of the follow-up period, 3 months (90 days) following discharge, the likelihood of drop-out by patients is minimal. However, as participants will be followed up in the community post-discharge, there is a potential for loss to follow-up. Participants who agree to complete the study questionnaires will be asked to provide contact information to be contacted at the 3 months follow-up time point—telephone, postal address and/or email. Patients who do not respond to contact requests to complete follow-up questionnaires by telephone will be considered as lost to follow-up for the purposes of questionnaire completion. However, routine clinical data will still be collected from patient records. Data will be retained for analysis.

#### Data management

Data will be entered under the participant’s ID onto the central database stored on NCTU servers. Access to the database will be via unique, individually assigned usernames and passwords, and only accessible to members of the CHARMER research team NCTU and external regulators if requested. The servers are protected by firewalls and are patched and maintained according to best practice. The physical location of the servers is protected physically and environmentally in accordance with University of East Anglia’s General Information Security Policy 3 (GISP3: Physical and environmental security).

The database has been developed by NCTU Data Management using REDCap,^21^ in conjunction with the CHARMER trial team. The database software provides several features to help maintain data quality, including maintaining an audit trail, allowing custom validations on all data, allowing users to raise data query requests and data quality rules to identify validation failure/missing data.

After completion of the study, the database will be retained on the servers of NCTU for ongoing analysis of secondary outcomes.

#### Confidentiality

All data will be sent to the Clinical Trials Unit which will be responsible for data analysis. Data will be stored in password protected computers and only shared in an anonymous format with the wider research team.

### Statistical methods for primary and secondary outcomes

#### Characterising and comparing sites

Participating hospitals and geriatricians and pharmacists will be characterised using descriptive statistics from the hospital site profile questionnaire and practitioner participant demographic questionnaire. Data will be visually compared with data captured from NHS Improvement’s hospital provider level benchmarking tool ‘model hospital’^22^ regarding the number and cost of staff on medicine for older people’s medicine wards. Demographic characteristics of patients between all study wards of the participating hospitals will be visually compared to explore whether they are comparable. For the sample of patients in the study cohort from whom consent is obtained either from the patient or consultee for additional non-routine data to be collected, the demographic characteristics of this group will be visually compared with the wider study cohort. This will establish whether the sub-set of patients for whom additional data are collected is a good representation of the patient cohort in the study.

#### Main effectiveness analysis

The primary analysis will be using intention-to-treat with a pre-specified statistical analysis plan (SAP) agreed prior to analysis. Analysis will be at the patient level and include data from all patients.

Unplanned readmission at 90 days, primary outcome measure, will be compared between the treatment and control groups using a logistic mixed regression model with hospital included as a random effect, and fixed factors will include randomisation arm and time period. If a significant number have more than one readmission, then a Poisson or Negative binomial model will be used as a secondary analysis. The assumptions of the models will be checked and, if appropriate, a Generalised Estimating Equation approach will be used if the random effect is not normally distributed, or a non-parametric bootstrap approach will be used to account for missing data.

#### Interim analyses

No interim analyses will be performed as the primary outcome measure will only be available once the trial is complete. The intervention is behavioural and focused on healthcare professionals; consequently, it was believed not to be necessary to access the primary outcome data, relating to rehospitalisation, until the trial was completed.

#### Additional analyses

A sensitivity analysis will adjust for relevant baseline characteristics, that is, age and the number of pre-admission medicines.

The number of medications stopped during the enhanced data collection period will be compared between the control and intervention using a linear mixed model including hospital as a random effect, and fixed factors will include randomisation arm and time period.

The proportion of patients per arm where a pre-admission medicine is proactively deprescribed will be compared using logistic regression, the same model as above.

The QoL measures will be compared between the control and intervention using a linear mixed model including hospital as a random effect, and fixed factors will include randomisation arm and time period.

#### Handling missing data

Due to the use of routinely collected data, it is unlikely that there will be significant missing data for the main analysis. If appropriate, we will use imputation to account for missing data for other non-routine outcomes.

A full SAP will be written prior to the start of any data analysis. As the SAP will be more detailed, any discrepancies in the analysis between the SAP and the protocol, the SAP will take priority.

### Health economics

A within-trial economic evaluation will be undertaken from the NHS perspective to estimate whether deprescribing is cost-effective.

Resources associated with providing the intervention will be included in the evaluation. This will include staff time required for activities such as attending workshops, watching video material, attending briefings, preparing an action plan and preparing benchmarking reports. The intervention may have an impact on resource use associated with NHS inpatient admissions in the follow-up period, as well as medicines use, which will also be investigated. For the consented sub-sample, data relating to Health Related Quality of Life and EuroQol 5-Dimension 5-Level (HRQoL (EQ-5D-5L)) and primary care contacts will also be collected.

Resource use data collection has been informed by lessons learnt from the feasibility study. The occurrence of activities related to the intervention, such as attendance at workshops, will be recorded by means of the fidelity framework conducted as part of the process evaluation. The protocol for the process evaluation will be reported separately.

The resource implications of these events will be costed by means of information obtained from the fidelity framework as well as additional insights gained from aspects of the feasibility study, for example, qualitative research. Where necessary, we will elicit further information required to cost the intervention. For example, eliciting expert opinion on the typical time required for deprescribing activities.

In terms of NHS resource use, data will be obtained from NHS England related to inpatient admissions and prescribing data. Additionally, data relating to the initial (recruitment) inpatient stay will be obtained from study sites. The feasibility study indicated that primary care-related resource use would be difficult to collect by the methods originally planned, namely either interviews with General Practitioners (GPs)/other primary care members with a prescribing role or general practice patient records review, without adding substantially to the burden of the study. For this reason, a bespoke section was added to the drug adverse event questionnaire to collect details of the participating patient’s participant primary care use at the 90-day follow-up telephone interview. The proposed questions have been shared with the trial management team and PPI members for comment. These data would be collected from the consented sub-study only. Resources identified will be combined with appropriate unit costs data, for example, NHS reference costs,^11^ to estimate the mean overall cost in each study arm. This will enable an estimate of any differences in the cost of the de-prescribing group compared with usual care.

The primary health economics analysis will be a cost-effectiveness study using the study’s primary outcome of re-admissions in the 90-day follow-up period. As part of the trial, we will collect measures of HRQoL using EQ-5D-5L.^23^ These will be collected in the consented sub-sample at baseline and at 90 days post-discharge, either by self-report or proxy response. This measure can be used to estimate quality adjusted life years (QALY), enabling the mean difference in QALY scores to be estimated (incremental effect). This will be carried out for the consented sub-sample and will be presented as a sensitivity analysis. As part of the analysis, we will explore whether it is sensible to use these values to impute QALY scores for the whole sample. If carried out, this will also be presented as an additional sensitivity analysis.

If the intervention arm is both less expensive, for example, due to lower medication or readmission costs, and more effective than usual care in terms of the primary outcome of readmissions at 90 days, then it would be said to dominate usual care and would be the preferred option. If neither the intervention nor the usual care is clearly preferred, we will estimate incremental cost-effectiveness ratios in terms of cost per re-admission prevented.^12^ The associated level of uncertainty will be characterised by estimating cost-effectiveness acceptability curves. Sensitivity analysis will also be undertaken to assess the robustness of results to changes in key assumptions. In line with the outcome analysis, all analyses will be conducted on an intention-to-treat basis. Appropriate actions will be taken where there is missing data, for example, imputing data by means of multiple imputation. As part of the health economic analysis, a health economics analysis plan will be written, in consultation with the CI and trial statistician and shared with team members.

A health economic model will be constructed to explore the effects of long-term changes in prescribing costs and mortality. The exact form of this model will be established in consultation with other members of the research team, as a clinical input to this process would be vital. A priori, this would be expected to take the form of a Markov model estimating cost per QALY.

### Data sharing

The protocol will be available on the NIHR website and within the ISRCTN register. The datasets generated during and/or analysed during the current study are not expected to be made available due to data sharing restrictions as part of section 251 approval and NHS England governance requirements. Statistical code will be available on request from allan.clark@uea.ac.uk.

### Oversight and monitoring

NCTU is responsible for managing the trial including operational delivery, data management, statistical analysis, health economics and quality assurance.

The Trial Steering Committee responsible for overseeing the CHARMER research programme will take on the responsibilities of the Trial Steering Committee and Data Management Committee at the beginning of the trial. The committee consists of a research-active consultant geriatrician (Chair), national lead for provision of clinical pharmacy services, senior medical statistician, senior clinical trialist, senior behavioural scientist and two PPI members, who are all independent of the sponsor and have no competing interests. The Committee was convened to meet approximately 6 monthly at key timepoints. Prof Bhattacharya (Co-CI and award holder) and Clark (Trial Statistician) are non-independent members. At least two representatives from NCTU, the funding award body, sponsor and trial team attend as observers reporting on progress and advising as requested.

As the primary outcome (readmissions to hospital) is obtained from routine data retained by NHS Digital and would not be available to the research team until after the last patient has completed, it was not possible to review the safety of the intervention during the trial.

### Adverse event reporting and harms

Adverse events are not being collected for this trial. Potential unintended harms arising from the intervention with respect to deprescribing discussions or the stopping or reducing of medications will be assessed by questionnaire in those participants who consent or for whom consultee assent is provided in the post-intervention enhanced data collection period. Harms identified will be reviewed by clinician members of the trial management group, categorised and reported as part of the trial publication. Complaints from participants, consultees, relatives or staff relating to the trial conduct will be directed to the Sponsor. NCTU will lead on investigating complaints in conjunction with the Trial Management Groups and Sponsor.

### Frequency and plans for auditing trial conduct

The delivery of the trial is overseen by the NCTU Quality Assurance team (who are independent of the research team and Sponsor) on a quarterly basis. Should issues arise, a risk-based audit may be undertaken at site or coordinating centre level.

### Communication of protocol amendments

Ethics (and Confidential Advisory Group) approval will be sought for all protocol amendments. Approved amendments will be communicated to participating sites including investigators, and where necessary, trial participants. Standard Protocol Items: Recommendations for Interventional Trials (SPIRIT) guidance: Plans for communicating important protocol modifications (eg, changes to eligibility criteria, outcomes, analyses) to relevant parties (eg, investigators, Research Ethics Committee/Institutional Review Boards (REC/IRBs), trial participants, trial registries, journals, regulators).)

### Ethical review

Ethical approval for the study was obtained from Wales REC2 on 4 July 2023, REF: 23/WA/0184. Confidential Advisory Group approval (REF: 23/CAG/0073) was obtained on 6 July 2023 to enable collection of anonymised routinely collected data for all participants during the non-enhanced data collection periods.

### Dissemination plans

The research team will follow the Guide to Disseminating Research (GuiDir) framework, which includes identification of target audiences for dissemination from the outset, agreeing dissemination goals, developing a dissemination plan, choosing appropriate dissemination channels and tracking the impact of the dissemination.^24^ Depending on the trial result, the goal would ultimately be national implementation of intervention and consequently target audiences will include service commissioners and representative bodies for the different actors within the trial.
