# Supplementary material for: Evaluation of the CompreHensive geriAtRician-led MEdication Review (CHARMER) deprescribing intervention in hospital: protocol for a cluster randomised stepped-wedge trial
Source: BMJ Open. 2026 Mar 18;16(3):e107396. doi: 10.1136/bmjopen-2025-107396 (PMC13007063; doi:10.1136/bmjopen-2025-107396)
Supplement: Supplementary file 1 [file bmjopen-2025-107396.reviewer_comments.pdf]

# PEER REVIEW HISTORY

BMJ Open publishes all reviews undertaken for accepted manuscripts. Reviewers are asked to complete a checklist review form and are provided with free text boxes to elaborate on their assessment. These free text comments are reproduced below.

**ARTICLE DETAILS**

**Title (Provisional)**

Evaluation of the CompreHensive geriAtRician-led MEdication Review (CHARMER) Deprescribing Intervention in Hospital: Protocol for a Cluster Randomised Stepped-Wedge Trial

**Authors**

Wright, David John; Alldred, David Phillip; Scott, Sion; Atkins, Bethany; Clark, Allan B; Colles, Antony; Hammond, Amber; Jones, Charlotte EL; Martin-Kerry, Jacqueline M; Patel, Martyn; Sims, Erika; Turner, David; Witham, Miles; Bhattacharya, Debi

---

This article was not externally reviewed at BMJ Open. Protocols that have been independently assessed prior to submission to BMJ Open are usually fast-tracked to publication on the grounds that further substantial changes will not be possible. This independent assessment will usually be external, independent review for both a substantial grant award from a non-commercial or government funder and ethics approval.
